# Maternal Characteristics, First-Trimester Biomarkers and Cell-Free DNA Fetal Fraction: A Single-Center NIPT Cohort over Ten Years

**DOI:** 10.3390/life16101677

**Published:** 2026-10-07

**Authors:** Maria Cezara Mureșan, Marius Bogdan Mureșan, Dan Dumitrașcu Biriș, Daniela-Eugenia Popescu

**Affiliations:** 1 Department of Obstetrics and Gynecology, Faculty of Medicine, Victor Babeș University of Medicine and Pharmacy, 300041 Timișoara, Romania; 2 Maternal-Fetal Medicine Center, 300331 Timișoara, Romania

**Keywords:** cell-free DNA, fetal fraction, non-invasive prenatal testing, PAPP-A, free β-hCG, body mass index, in vitro fertilization, first-trimester screening, test failure

## Abstract

The fetal fraction (FF) of cell-free DNA determines the reliability of non-invasive prenatal testing (NIPT); whether first-trimester placental biomarkers add information beyond maternal factors is unclear. We linked NIPT samples from a single fetal medicine center over ten years (1162 pregnancies) to first-trimester screening records and modeled log FF with hierarchical regression (maternal/technical covariates → PAPP-A → free β-hCG), cluster-robust standard errors, and an order-independent bootstrapped partition of the biomarker contribution. In the primary complete-case model (*n* = 292), FF was 3.4% lower per kg/m^2^ of BMI (95% CI −4.4 to −2.3) and 24% lower after IVF/ICSI. Free β-hCG was independently associated with FF (+11.0% per doubling of MoM, 95% CI +5.3 to +16.9; semi-partial R^2^ = 0.046), whereas the PAPP-A association was weaker and less consistent (+4.8%, 95% CI −0.6 to +10.4; semi-partial R^2^ = 0.010). The β-hCG association persisted after excluding high-risk NIPT calls, documented multiple gestation and IVF/ICSI, within platform-restricted subsets and with a restricted documentation-to-draw interval. No independent association with gestational age was identified in the primary model; low-FF and no-call results were too few for adjusted inference. Free β-hCG carries independent information on FF, consistent with, but not proof of, a link to syncytiotrophoblast biology.

## 1. Introduction

Cell-free DNA (cfDNA) screening for the common autosomal trisomies has become an integral part of prenatal care since its introduction in 2011, with detection rates above 99% for trisomy 21 and false-positive rates below 0.1% in meta-analyses [1,2]. The fetal component of maternal plasma cfDNA originates predominantly from apoptotic syncytiotrophoblast [3,4], and its proportion, the fetal fraction (FF), is the main analytical determinant of test reliability: a low FF reduces the statistical separation between euploid and aneuploid samples and, below a platform-specific threshold (usually 2–4%), leads to a failed or “no-call” result [5,6]. Test failure is not a neutral event. It delays reassurance or diagnosis, often requires a second sample, and has been repeatedly associated with an increased prevalence of fetal aneuploidy, in particular trisomies 18 and 13 and triploidy, as well as with placenta-mediated complications [7,8,9].

The determinants of FF have been described in large laboratory series. Maternal weight or body mass index (BMI) is the strongest and most consistent factor, with FF decreasing as maternal weight increases, presumably through dilution by maternal cfDNA released from an expanded adipose and inflammatory compartment [10,11,12,13]. FF rises slowly with gestational age, is lower in pregnancies conceived by assisted reproduction [14,15], and is altered by fetal karyotype, being higher in trisomy 21 and lower in trisomies 18 and 13 [16]. Fewer studies have addressed whether the FF also reflects placental function as measured by the biochemical markers of the first-trimester combined test. In the original Fetal Medicine Foundation series, FF at 11–13 weeks increased with serum PAPP-A and free β-hCG [10], and later work reported that a low FF is associated with first-trimester markers of impaired placentation, including low PAPP-A and placental growth factor, and with subsequent pre-eclampsia and fetal growth restriction [17,18,19]. These observations have led to the hypothesis that FF is not merely a technical quantity but partly an index of early trophoblast mass or turnover. The question has practical relevance, because it would imply that pregnancies with abnormal first-trimester biochemistry are more likely to fail cfDNA testing and, conversely, that FF could add information to placental risk assessment.

Most published cohorts on FF come from testing laboratories, with limited maternal phenotyping and no direct access to first-trimester ultrasound and biochemistry data. Clinical cohorts in which NIPT results can be linked at the pregnancy level to a complete first-trimester screening record are comparatively rare, and they offer a different vantage point: the maternal characteristics, PAPP-A and free β-hCG multiples of the median (MoM), nuchal translucency (NT) and the combined-test risk were obtained as part of the first-trimester screening assessment, in the same clinical setting, and could be linked to the NIPT record. Such cohorts also capture the real-world heterogeneity of platforms and of gestational age at sampling that characterizes NIPT use outside centralized programs.

The primary aim of this study was to determine whether first-trimester biomarkers—PAPP-A and free β-hCG—are independently associated with cfDNA FF after adjustment for the established maternal and technical determinants, and how much variance in FF they explain beyond these factors. Secondary aims were to describe low FF and no-call results in relation to maternal characteristics and biomarkers, to describe the relationship between prior combined-test risk and NIPT results, and to document the evolution of NIPT use in the center over ten years. The design was deliberately agnostic: a null result for PAPP-A would be informative, indicating limited independent overlap between FF and first-trimester placental biochemistry after accounting for maternal and technical factors.

## 2. Materials and Methods

### 2.1. Study Design and Setting

This was a retrospective cohort study conducted at a tertiary fetal medicine center in western Romania. The center performs first-trimester combined screening according to Fetal Medicine Foundation (FMF) protocols, and since 2015 has offered cfDNA testing either as a second-tier test after combined screening or as a primary test at the woman’s request. Blood samples for NIPT were drawn in the center and shipped to external laboratories; from 2023, a single-nucleotide polymorphism (SNP)-based assay became available locally. The study was approved by the Ethics Committee of the Maternal-Fetal Medicine Center, Timișoara (framework approval No. 2/2026; initial approval No. 1/2026); the requirement for individual informed consent was waived because of the retrospective, non-interventional design and the use of pseudonymized data.

### 2.2. Data Sources and Linkage

All cfDNA test records entered into the center’s ultrasound and screening database (Astraia, Astraia Software GmbH, Munich, Germany) between 2015 and 2025 were exported, together with the first- and second-trimester examination tables. For each NIPT record, the following were retrieved: provider and assay, date of blood draw, gestational age at the examination at which the result was recorded, reported FF (%), results for trisomies 21, 18 and 13 (low risk, high risk, and no result), fetal sex and sex chromosome findings, and any subsequent rapid aneuploidy test (QF-PCR), karyotype and pregnancy outcome. NIPT records were linked to first-trimester examinations of the same pregnancy using the internal patient identifier and the pregnancy number. From the linked first-trimester record, we extracted maternal date of birth, weight and height, smoking, mode of conception, parity, crown–rump length (CRL), NT, serum PAPP-A and free β-hCG expressed as MoM (Kryptor, BRAHMS, Hennigsdorf, Germany), mean uterine artery pulsatility index MoM, and the adjusted risk for trisomy 21 from the combined test. When a pregnancy had more than one first-trimester examination, the examination with a CRL of 45–84 mm and complete biochemistry was retained.

Gestational age at blood draw was recalculated from the date of blood draw and the ultrasound-dated gestational age at the recording examination, because the gestational age stored with the NIPT record referred to the visit at which the result was entered (median 7 days, interquartile range [IQR] 0–50, after the draw). Values outside 9–40 weeks were treated as invalid for analysis and set to missing. Maternal BMI was calculated from first-trimester weight and height; values outside the prespecified plausibility range of 15–60 kg/m^2^ were treated as missing. Maternal age was calculated at the date of blood draw. All analyses were performed on a pseudonymized dataset in which the patient identifier was replaced by a salted hash and all direct identifiers were removed.

### 2.3. Analytical Populations

Analytical populations were defined a priori and are shown in the cohort flow diagram (Figure S1). The export contained 1197 NIPT records. Twenty-seven records were additional rows for the same blood draw (3 of them exact duplicates; in one case, two different FF values had been entered for the same draw and FF was set to missing), leaving 1170 distinct blood draws in 1162 pregnancies of 1146 women; a total of 35 pregnancies had more than one record, but only 8 had a true second draw on a later date. For all FF analyses, the first draw per pregnancy was used; repeat draws were counted descriptively. (i) The total NIPT cohort (*n* = 1162 pregnancies) was used to describe test use, results, platform characteristics and temporal trends. (ii) The linked cohort (*n* = 760) comprised pregnancies with a first-trimester examination in the center and was the population for the primary analysis; pregnancies without a linked record (*n* = 402) were retained in descriptive analyses and compared with the linked cohort. (iii) Within the linked cohort, nested complete-case populations were defined by the availability of the variables required by each analysis: FF reported (*n* = 714); FF with a valid gestational age at draw of 9–40 weeks (*n* = 707); the preceding plus a valid BMI (*n* = 502); the preceding plus the remaining baseline covariates, principally fetal sex (*n* = 467; baseline cohort); and the preceding plus both serum markers (*n* = 292; primary Model 3), of which 286 remained after exclusion of high-risk NIPT calls. The univariable BMI–FF analysis required FF and BMI (*n* = 505); biomarker analyses additionally used a valid gestational age (PAPP-A *n* = 316; free β-hCG *n* = 317; both markers with BMI *n* = 314). The linked cohort included 8 documented multiple pregnancies (5 in the primary model) and 43 IVF/ICSI conceptions among 759 with a documented mode of conception (25 in the primary model), of which 4 were with donor oocytes (none in the primary model); a total of 6 pregnancies in the primary model had a high-risk NIPT call for trisomy 21, 18, or 13. Conception subtype was recorded in only 29 linked pregnancies; absence of a donor-oocyte code does not establish use of autologous oocytes. Absence of a multiple-gestation flag was the operational singleton definition. These subgroups were handled in sensitivity analyses (Section 2.5). Because the mode of conception was recorded as documented IVF/ICSI or not, the reference category is “no documented IVF/ICSI” rather than spontaneous conception.

### 2.4. Outcomes

The primary outcome was the FF as a continuous variable, log-transformed because of its right-skewed distribution; effects are reported as the percentage change in FF per unit of a covariate or per doubling of a biomarker MoM. A continuous outcome was preferred to a dichotomous “low FF” because the reporting threshold for test failure differs between platforms. Secondary outcomes, described without adjusted modeling because of the small numbers of events, were (a) low FF, defined as FF < 4% as reported by the laboratory; (b) a no-call result, defined as “no result” for at least one of the three trisomies in the absence of a high-risk call; and (c) a repeat draw in the same pregnancy.

### 2.5. Statistical Analysis

Continuous variables are summarized as median (IQR) and categorical variables as counts (%). Univariable associations with FF were summarized with Spearman’s rank correlation; platform distributions and rare outcomes are described without inferential comparisons.

The primary analysis used three hierarchical linear regression models for log FF in the complete-case linked cohort (*n* = 292). Model 1 included maternal BMI, gestational age at blood draw, maternal age, smoking, documented IVF/ICSI, fetal sex, and platform (Natera Panorama, locally processed Panorama, Veracity, NIFTY, Harmony, NIPS, and other). Model 2 added log PAPP-A MoM and Model 3 added log free β-hCG MoM. Because the increment in R^2^ of a sequential model depends on the order of entry, both orders of entry are reported and the contribution of each biomarker was also quantified in an order-independent manner: the semi-partial R^2^ (the decrease in R^2^ when the biomarker is removed from the full model, i.e., its unique contribution given all other terms), the partial R^2^ (the semi-partial R^2^ divided by 1 − R^2^ of the model without that biomarker), and the joint contribution of both biomarkers (R^2^ of Model 3 minus R^2^ of Model 1). Confidence intervals for the joint, semi-partial, and partial R^2^ were obtained by percentile cluster bootstrap (2.5th–97.5th percentiles) with 5000 replicates per model, resampling women with replacement, with a fixed seed. Standard errors used woman-level clustering with finite-sample correction and t-based inference. The primary model included 289 women: 286 with one pregnancy and 3 with two. All nested models within a cohort used the same observations. Bootstrap intervals for non-negative R^2^ increments are not null-hypothesis tests.

Missing covariates were handled by complete-case analysis. Both serum biomarkers were available in 338 of 760 linked pregnancies and unavailable as a pair in 422 (55.5%). Referral pathways and screening outside the center may contribute to missingness, but the available records do not establish its mechanism. The multiple-imputation analysis included in the original submission was removed because its exact implementation could not be reproduced during the revision audit. We did not replace it with a newly specified imputation model, because the two exposures of primary interest were jointly missing in more than half of the linked cohort and any new imputation analysis would therefore rely on substantial, untestable assumptions. We do not consider complete-case analysis inherently preferable under uncertain missingness; it remains susceptible to selection bias. We therefore characterized selection directly by comparing pregnancies with and without both biomarkers (Table S1) and interpret the biomarker estimates as applying to women who underwent both combined screening and NIPT in the center.

Sensitivity analyses of Model 3 (pre-specified, or added during peer review) were: (a) exclusion of the 6 pregnancies with a high-risk NIPT call for trisomy 21, 18 or 13, because fetal aneuploidy alters FF; (b) exclusion of documented multiple pregnancies; (c) exclusion of documented IVF/ICSI conceptions; (d) exclusion of NIFTY samples, whose FF distribution differed markedly from the other platforms; (e) restriction to SNP-based assays (Natera and locally processed Panorama); (f) exclusion of high-risk NIPT calls combined with (d) or (e); and (g) restriction to pregnancies in which the first-trimester examination was documented on or before the NIPT draw, and to those in which it was documented 0–28 days before or on the day of the draw. The relationship between FF and gestational age at draw was examined further with the full Model 3 within gestational windows (10–14, 11–13^+6^, 10–20, >14–20 and >20–30 weeks), with a model allowing separate slopes before and after 14 weeks, and with the Model 1 covariates in the baseline cohort within the 10–14-week window, with and without high-risk calls.

Low FF and no-call results were described by BMI category and mode of conception with counts and proportions. Adjusted logistic models were not fitted because the numbers of events were too small to support multivariable inference; these outcomes are reported descriptively and as hypothesis-generating.

For the screening analyses, NIPT results were tabulated by category of the prior combined-test risk for trisomy 21, and the proportion of high-risk NIPT calls that would have been selected by a contingent strategy offering NIPT only at or above a risk threshold (1 in 100, 1 in 300, and 1 in 1000) was calculated among pregnancies with a known combined-test risk. Because confirmatory testing was documented in a minority of pregnancies, these figures refer to high-risk NIPT calls, not to confirmed aneuploidies. Temporal trends (2016–2025) in test volume, platform, gestational age at sampling, prior screening, and high-risk call rate were described; no formal trend test is reported. Analyses were performed in Python 3.12 (pandas 2.2.3, NumPy 2.3.5, and statsmodels 0.15.0), and all analytical denominators, descriptive counts, and model estimates were re-derived from a single frozen analytic dataset in a reproducibility audit before resubmission. A two-sided *p* < 0.05 was considered statistically significant; no correction for multiple testing was applied, and secondary analyses should be interpreted as hypothesis-generating.

## 3. Results

### 3.1. Study Population

Between November 2015 and December 2025, 1197 cfDNA records corresponding to 1170 distinct blood draws were recorded in 1162 pregnancies of 1146 women (Figure S1); eight pregnancies (0.7%) had a second draw on a later date, 35 (3.0%) had more than one record, and 16 women had NIPT in two pregnancies. Annual volume rose from 78 tests in 2016 to 256 in 2024 (Figure 1A). An FF was reported for 1085 first draws (93.4%). Gestational age at blood draw was known for 1146 pregnancies and had a median of 13.6 weeks (IQR 11.9–18.0); a total of 639 samples (55.8%) were drawn at ≤14 weeks, 260 (22.7%) between 14 and 20 weeks, and 247 (21.6%) after 20 weeks. The median FF was 9.1% (IQR 6.6–12.0); a total of 45 samples (4.1%) had FF < 4%.

A high-risk NIPT call for at least one trisomy was reported in 52 pregnancies (4.5%): trisomy 21 in 38 (3.3%), trisomy 18 in 8 (0.7%), and trisomy 13 in 6 (0.5%). A no-call result without a high-risk call occurred in 12 pregnancies (1.0%). Only eight pregnancies had a second draw on a later date; additional records on the same date were not classified as redraws.

Seven hundred and sixty pregnancies (65.4%) had a first-trimester examination in the center and formed the linked cohort (Table 1). Compared with the 402 unlinked pregnancies, which were referred for NIPT or for second-trimester ultrasound without prior screening in the center, linked pregnancies were sampled earlier (median 13.3 versus 14.9 weeks) and had similar FF (9.1% versus 8.8%), high-risk call rates (5.0% versus 3.5%) and no-call rates (1.3% versus 0.5%). In the linked cohort, maternal weight was recorded in 541 pregnancies, and a valid BMI was available in 539 (70.9%), and first-trimester biochemistry in 338 (44.5%). Of the 714 linked pregnancies with a reported FF, 707 had a valid gestational age at draw, 502 also had a valid BMI, 467 had complete baseline covariates, and 292 additionally had both serum markers and formed the population of the primary model (Figure S1). Linked pregnancies with and without serum biochemistry are compared in Table S1: those with biochemistry were slightly older (median 34.3 versus 33.7 years), were sampled later (13.7 versus 12.9 weeks), were more often conceived by IVF/ICSI (8.3% versus 3.6%) and less often had a high-risk NIPT call (3.0% versus 6.6%), whereas BMI (22.9 versus 23.0 kg/m^2^) and FF (9.6% versus 8.9%) were similar. Among the 292 pregnancies in the primary model, the median interval between the date of the first-trimester examination and the NIPT draw was 3 days (IQR 0–19; range −28 to +148 days); the examination was documented before the draw in 168, on the same day in 60 and after the draw in 64 pregnancies, so that in 228 (78.1%) it was documented on or before the draw and in 179 (61.3%) within 0–28 days before or on the day of the draw. The date available is the date of the first-trimester examination at which the biochemistry was documented, not a separately verified date of serum sampling.

### 3.2. Platforms and Fetal Fraction Reporting

Platforms are summarized in Table 2 using the same seven categories as the regression models. SNP-based Panorama testing accounted for 467 of 1162 pregnancies (40.2%). FF was reported in 87.3–98.2% of first draws across platform categories. NIFTY had a higher median FF (10.6%) than the two SNP-based categories (8.1–8.6%). Ten of the 12 no-call results occurred with SNP-based assays. These descriptive differences support adjustment for platform and sensitivity analyses within restricted platform groups, but do not establish uniform FF measurement across laboratories.

### 3.3. Univariable Associations with Fetal Fraction

In the linked cohort, the unadjusted BMI–FF correlation was negative (Spearman ρ = −0.32, *n* = 505). In the baseline cohort, median FF decreased across BMI categories from 11.5% for BMI < 18.5 kg/m^2^ to 6.1% for BMI ≥ 35 kg/m^2^ (Table 3). Among pregnancies with FF, valid gestational age and the relevant marker, the Spearman correlations were 0.21 for free β-hCG (*n* = 317) and 0.12 for PAPP-A (*n* = 316). Across 9–40 weeks, the audited gestational-age correlation was ρ = 0.035 (*n* = 707, *p* = 0.354). Figure 2 displays the unadjusted BMI and biomarker relationships; the adjusted biomarker estimates are reported in Table 4.

### 3.4. Multivariable Determinants of Fetal Fraction

The hierarchical models are summarized in Table 4, and the fully adjusted estimates are shown in Figure 3. Model 1 (maternal and technical covariates; *n* = 292) explained 18.0% of the variance in log FF (R^2^ = 0.180). In the fully adjusted Model 3 (R^2^ = 0.238), FF was 3.4% lower per kg/m^2^ of BMI (95% CI −4.4 to −2.3) and 23.8% lower in pregnancies with documented IVF/ICSI (95% CI −35.3 to −10.3), whereas gestational age at blood draw showed no independent association (−0.2% per week, 95% CI −1.4 to +1.1; *p* = 0.81); maternal age, smoking and fetal sex were included as covariates, and platform contributed modestly.

Adding PAPP-A after Model 1 increased R^2^ by 0.011, and adding free β-hCG thereafter by a further 0.046; in the reciprocal order, β-hCG first increased R^2^ by 0.048 and PAPP-A thereafter by 0.010. In Model 3, FF was 11.0% higher per doubling of β-hCG MoM (95% CI +5.3 to +16.9; *p* < 0.001), while the PAPP-A estimate was smaller and not statistically significant (+4.8% per doubling, 95% CI −0.6 to +10.4; *p* = 0.082). In the order-independent partition, the two biomarkers jointly explained 5.7% of the variance in log FF beyond the maternal and technical covariates (joint ΔR^2^ = 0.057, 95% bootstrap CI 0.017–0.121); the unique (semi-partial) contribution of β-hCG was 0.046 (95% CI 0.011–0.098) and that of PAPP-A 0.010 (95% CI 0.000–0.041), with partial R^2^ of 0.057 (95% CI 0.014–0.126) and 0.012 (95% CI 0.000–0.056), respectively. The unique contribution of β-hCG was therefore about five times that of PAPP-A, and this ranking did not depend on the order in which the biomarkers were entered.

### 3.5. Sensitivity and Gestational-Age Analyses

The β-hCG association persisted in all sensitivity analyses reported in Table 5. After exclusion of the six pregnancies with a high-risk NIPT call for trisomy 21, 18 or 13 (*n* = 286), FF was 11.6% higher per doubling of β-hCG MoM (95% CI +5.9 to +17.7; *p* < 0.001) and the PAPP-A coefficient increased slightly and became nominally significant (+6.4%, 95% CI +1.0 to +12.1; *p* = 0.020); R^2^ rose from 0.185 (Model 1) to 0.252 (Model 3), the joint contribution of the two biomarkers was 0.067 (95% CI 0.023–0.130), with semi-partial R^2^ of 0.051 (95% CI 0.014–0.103) for β-hCG and 0.016 (95% CI 0.000–0.048) for PAPP-A. Exclusion of the five documented multiple pregnancies (*n* = 287) or of the 25 documented IVF/ICSI conceptions (*n* = 267) did not change the β-hCG estimate (+10.8% and +12.9% per doubling), whereas PAPP-A was of borderline significance in singletons (+5.3%, *p* = 0.052). In platform-restricted subsets, the β-hCG estimate was +10.4% after excluding NIFTY (*n* = 252) and +8.6% in SNP-based assays alone (*n* = 140), and +11.2% and +8.5% when high-risk calls were also excluded, while the PAPP-A coefficient fell to +1.4% to +3.4% with confidence intervals including zero in every platform-restricted analysis. Restriction to pregnancies in which the first-trimester examination was documented on or before the NIPT draw (*n* = 228) or 0–28 days before or on the day of the draw (*n* = 179) gave β-hCG estimates of +12.5% and +13.2% per doubling; the PAPP-A coefficient was +3.1% (*p* = 0.32) and +1.1% (*p* = 0.74), respectively.

Because no association between FF and gestational age at draw was detected in the primary model, the relationship was examined further (Table S2). Across the whole 9–40-week range, FF was not correlated with gestational age (ρ = 0.03, *n* = 707, *p* = 0.35), and the joint test of the gestational-age terms in a model allowing separate slopes before and after 14 weeks did not identify an overall association (Wald *p* = 0.845). Within gestational windows, the fully adjusted Model 3 gave no association in any window (10–14 weeks, *n* = 155: −1.1% per week, 95% CI −7.1 to +5.2; 11–13^+6^ weeks, *n* = 128: −3.8%; 10–20 weeks, *n* = 263: −1.2%; >14–20 weeks, *n* = 108: −1.0%; >20–30 weeks, *n* = 26: +5.5%). In the larger baseline cohort without the biochemistry requirement, however, FF increased with gestational age within the 10–14-week window after adjustment for BMI, maternal age, smoking, IVF/ICSI, fetal sex and platform (*n* = 283: +5.9% per week, 95% CI +1.8 to +10.1; *p* = 0.004), and after additionally excluding high-risk calls (*n* = 268: +5.3% per week, 95% CI +1.0 to +9.7; *p* = 0.014); when this baseline model was restricted to the same 155 pregnancies as the biochemistry model, the coefficient was +0.5% per week (*p* = 0.88), indicating that the difference reflects the population rather than the covariates.

### 3.6. Low Fetal Fraction and No-Call Results

In the linked cohort with FF, a valid BMI and gestational age (*n* = 502), FF < 4% occurred in 20 pregnancies (4.0%): 9 of 53 (17.0%) with BMI ≥ 30 kg/m^2^ versus 11 of 449 (2.4%) with BMI < 30 and 4 of 35 (11.4%) with documented IVF/ICSI versus 16 of 467 (3.4%) with no documented IVF/ICSI. A no-call result occurred in 10 of 760 linked pregnancies (1.3%; Table 1). Among the 316 of 338 linked pregnancies with both serum biomarkers and an available FF measurement, 10 (3.2%) had FF < 4%. Six no-call results occurred among the 338 pregnancies with both biomarkers. These small event counts were not used for adjusted biomarker inference.

Low-FF and no-call results are distinct outcomes: a laboratory may issue a result at FF < 4%, and a no-call can occur for reasons other than low FF.

### 3.7. Prior Combined-Test Risk and NIPT Results

A combined-test risk for trisomy 21 was available for 902 pregnancies (77.6% of the total cohort), including 49 with a risk of 1 in 100 or higher (5.4%). Table 6 shows 18 high-risk T21 calls and 21 high-risk calls for any of the three trisomies among these 49 pregnancies; a total of 31 had no high-risk T21 call, while 28 had no high-risk call for any of the three trisomies. None had a no-call result. Among the 853 pregnancies with a combined-test risk below 1 in 100, 11 had a high-risk T21 call and 17 had a high-risk call for any trisomy. A contingent threshold of 1 in 100 would have selected 18 of the 29 high-risk T21 calls with a known combined-test risk (62.1%); thresholds of 1 in 300 and 1 in 1000 would have selected 22 (75.9%) and 24 (82.8%), respectively, while selecting 5.4%, 12.9%, and 27.4% of pregnancies with a known risk. These proportions concern screening calls, not confirmed aneuploidies.

Diagnostic confirmation and pregnancy outcomes were incompletely documented. Consequently, this cohort does not support reliable estimates of NIPT positive predictive value or diagnostic sensitivity.

### 3.8. Temporal Trends

By year of the first NIPT blood draw, annual volume increased from 78 pregnancies in 2016 to 256 in 2024 (Figure 1). The proportion with a prior T1 record in the center declined from 85.9% in 2016 to 49.2% in 2024, then was 63.0% in 2025. The high-risk NIPT call rate was 6.4% in 2016, reached 10.5% in 2021, and was 2.0%, 2.0% and 2.4% in 2023, 2024 and 2025, respectively. Figure 1 presents descriptive trends; these data do not establish the reasons for changes in testing pathways.

## 4. Discussion

### 4.1. Principal Findings

In this single-center cohort of 1162 pregnancies tested over ten years, in which NIPT results were linked to the first-trimester screening record of the same pregnancy, the FF of cfDNA was independently associated with maternal BMI and with documented IVF/ICSI, with an additional, moderate contribution of the testing platform. Of the two serum markers of the combined test, free β-hCG was independently and consistently associated with FF, whereas the association with PAPP-A was weaker and less consistent: after adjustment, a doubling of β-hCG MoM corresponded to an 8.5–13.2% higher FF across all sensitivity analyses and uniquely explained about 5% of the variance beyond maternal and technical factors, while PAPP-A uniquely explained about 1%, with a coefficient about half that of β-hCG that reached nominal significance only after exclusion of high-risk calls and was compatible with no effect in every platform-restricted and timing-restricted subset. The ranking of the two biomarkers did not depend on the order of entry into the models. The β-hCG association remained consistent after exclusion of pregnancies with a high-risk NIPT call, documented multiple gestation, and documented IVF/ICSI, in platform-restricted analyses, and under restrictions on the interval between biochemistry documentation and the cfDNA draw. These sensitivity analyses support the robustness of the association but cannot exclude residual confounding or measurement error. No independent association between FF and gestational age was identified in the primary model, but FF increased with gestational age within the 10–14-week window in the larger baseline cohort. Low-FF and no-call results were too few for adjusted analysis; descriptively, low FF was more frequent with obesity and with IVF/ICSI. Finally, the cohort documents the transition of NIPT in this setting from a contingent second-tier test to a broadly used first-line test, with a fall in the high-risk call rate from approximately 6–11% in selected earlier years to approximately 2% in 2023–2025; a contingent strategy at a threshold of 1 in 100 would have selected less than two-thirds of the high-risk trisomy 21 calls.

### 4.2. Maternal and Technical Determinants

The magnitude of the BMI association (−3.4% of FF per kg/m^2^, i.e., a FF about 16% lower for every 5 kg/m^2^) and the higher proportion of FF < 4% in obese women (17% versus 2.4%) are consistent with the large laboratory series [10,11,12,13,20] and with the meta-analytic evidence that maternal weight is the dominant risk factor for low FF and test failure [6]. The lower FF after documented IVF/ICSI (−24%) and the higher proportion of FF < 4% agree with previous reports [14,15] and with the excess of failed results in assisted conceptions in the FMF experience [8]. The mechanism is not established; altered early placentation after embryo transfer, higher maternal cfDNA turnover in women undergoing ovarian stimulation, and confounding by the indication for treatment have all been proposed. Because the database records only documented IVF/ICSI, the reference group may include other forms of assisted conception, and the direction of any resulting bias cannot be established. Notably, no no-call result occurred in a pregnancy with documented IVF/ICSI, possibly because such pregnancies were preferentially tested with platforms that do not apply a FF threshold.

No independent association between gestational age at blood draw and FF was identified in the primary model, which spans 9–40 weeks; this differs from the slow rise in FF reported across the first and second trimesters in laboratory series [11,12]. The additional analyses (Table S2) clarify the discrepancy only in part. In the fully adjusted biochemistry model, no association was found in any gestational window, and the joint test of the two gestational-age terms in a model with separate slopes before and after 14 weeks was not statistically significant. In the larger baseline cohort without the biochemistry requirement, however, FF did increase with gestational age within the 10–14-week window after adjustment (about 6% per week, corresponding to roughly 0.5 percentage points per week at the median FF), in keeping with the published series, and this difference was attributable to the population rather than to the covariates, because the baseline model restricted to the same pregnancies as the biochemistry model also showed no association. Two features of the cohort are likely to contribute: later sampling was largely driven by referral of women without prior screening, often for an ultrasound finding, so that a linear term over 9–40 weeks averages a first-trimester rise with a selected later population, and the pregnancies with biochemistry were sampled within a narrow gestational range around the combined test, which leaves little variation in gestational age for the model to explain. The gestational-age coefficient of the primary model should therefore be read as “no independent linear association detected in this cohort” rather than as evidence that FF is unrelated to gestational age; importantly, the biomarker associations were unchanged when the interval between T1 examination/documentation and the draw was restricted. The platform effect deserves emphasis: FF is not a standardized quantity, and the distribution reported by NIFTY differed substantially from that of the SNP-based and other assays, as noted previously when comparing FF estimation methods [21]. This is why we treated platform as a covariate, used a continuous outcome, and repeated the analyses in platform-restricted subsets.

### 4.3. Free β-hCG, PAPP-A and the Placental Origin of Fetal Fraction

Both PAPP-A and free β-hCG are produced by the syncytiotrophoblast, the tissue from which the fetal component of maternal plasma cfDNA is released [3,4]. The original FMF description of FF at 11–13 weeks found that FF increased with both markers [10], and the hypothesis that a low FF reflects a small or dysfunctional placenta has been supported by the association of low FF with low PAPP-A and placental growth factor and with subsequent placental complications [17,18,19]. Our data refine this picture. The association with free β-hCG was consistent across all sensitivity analyses—including exclusion of high-risk NIPT calls, documented multiple pregnancies and IVF/ICSI conceptions, platform-restricted subsets, and restriction of the interval between the documentation of biochemistry and the draw—whereas the association with PAPP-A was smaller, reached nominal significance only after exclusion of high-risk calls, and was compatible with no effect in every platform-restricted and timing-restricted subset. Both markers came from the same clinical screening setting and were modeled together. However, examination/documentation dates do not verify serum-sampling dates, and differences in assay performance, biological variability, or measurement error cannot be excluded.

One interpretation, which we present as a hypothesis rather than a mechanism, is that FF may be more closely related to the endocrine activity, or the cellular turnover, of the syncytiotrophoblast than to the processes reflected by circulating PAPP-A. Free β-hCG is derived predominantly from placental trophoblast, and hCG signaling is closely linked to trophoblast differentiation and syncytialization [22,23,24]. PAPP-A is also predominantly placental in pregnancy, but circulating PAPP-A reflects a metalloprotease involved in local IGF bioavailability rather than a direct measure of syncytiotrophoblast turnover [25,26], and low first-trimester PAPP-A is a stronger marker of later placental insufficiency than of trophoblast volume [27]. If placental cfDNA release reflects syncytiotrophoblast turnover and cell death [3,4,28], a marker linked to syncytiotrophoblast biology could plausibly correlate with FF more closely than a marker of the invasive or metabolic phenotype of the placenta. The observation is also compatible with the higher FF in trisomy 21 pregnancies, in which free β-hCG is elevated, and with the lower FF in trisomies 18 and 13, in which both markers are reduced [16,29]; in our data, however, the β-hCG association remained similar after excluding pregnancies with a high-risk NIPT call, although undiagnosed aneuploidy among the remaining pregnancies cannot be excluded. Our study measured associations, not mechanisms: it cannot distinguish trophoblast mass, endocrine activity, and cellular turnover, and the variance uniquely explained by β-hCG, although consistent, was modest (about 5%).

The weaker and less consistent PAPP-A association does not establish that low PAPP-A has no relevance to cfDNA test performance. Only 10 low-FF and 6 no-call results occurred among linked pregnancies with both biomarkers; these counts are insufficient for reliable adjusted inference about rare outcomes. Whether very low PAPP-A identifies pregnancies at increased risk of test failure remains a hypothesis for larger cohorts with placental outcomes [8,9].

### 4.4. Test Failure and Screening Strategy

The overall rate of no-call results (1.0%) and of FF < 4% (4.1%) was at the low end of published figures [5,6], and no-call was concentrated in the SNP-based assays, which apply an explicit FF threshold, whereas other platforms reported results down to FF values of 1–2%. The clinical implication is that the same sample can be “failed” or “reported” depending on the laboratory, and that the interpretation of a very low FF is platform-dependent, because laboratories differ in FF estimation and in the minimum FF required for reporting [5]. Reporting of FF and transparent disclosure of laboratory-specific criteria for test failure are therefore important components of NIPT reporting; the American College of Medical Genetics and Genomics has specifically recommended visible reporting of FF and of the reason for a no-call result [30], and the 2025 Society for Maternal-Fetal Medicine Consult Series on cfDNA screening recommends genetic counseling, comprehensive ultrasound evaluation and the offer of diagnostic testing after a non-reportable result because of the increased risk of fetal aneuploidy, rather than simple repeat testing [31].

The screening data illustrate changing NIPT use in this center. In pregnancies with a known combined-test risk, a threshold of 1 in 100 would have selected 18 of 29 high-risk T21 calls (62.1%), leaving 11 of 29 (37.9%) unselected; a threshold of 1 in 1000 would have left 5 of 29 unselected. These figures concern high-risk calls, not confirmed aneuploidies, and do not measure diagnostic sensitivity because confirmation was incomplete and the tested population was selected. They describe the trade-off between the number selected for NIPT and subsequent high-risk calls within this cohort [32,33]. Among the 49 pregnancies with a high-risk combined test, 28 had no high-risk NIPT call for any of the three trisomies, illustrating the potential role of second-tier cfDNA screening [34].

### 4.5. Strengths and Limitations

The main strength of this study is the linkage of NIPT records to a complete first-trimester screening record obtained in the same center, so that maternal characteristics, serum markers measured in a single laboratory, NT, and the combined-test risk were available for the same pregnancy, documented at a median of 3 days from the cfDNA draw. The cohort also reflects real-world NIPT use, with multiple platforms and a wide range of gestational ages at sampling, and the analytical plan—a continuous primary outcome, hierarchical models with an order-independent partition of the biomarker contribution, cluster-robust inference, a 5000-replicate bootstrap, an extensive set of sensitivity analyses and a reproducibility audit of the analytical denominators and model estimates—was designed to be informative regardless of the direction of the PAPP-A result.

The limitations are those of a retrospective clinical database. A valid BMI was missing in 29% and serum biochemistry in 55% of the linked pregnancies, potentially reflecting screening outside the center and other referral-related factors, so that the complete-case primary model rests on 292 pregnancies, a quarter of the total cohort. The missingness mechanism could not be established from the available records, and the complete-case analysis is unbiased only if, given the covariates, completeness does not depend on FF; this assumption is untestable, so the complete-case estimates remain susceptible to selection bias. Multiple imputation would not have removed this limitation, because the missing variables were the exposures themselves, absent as a pair in more than half of the cohort, and would have been imputed under equally untestable assumptions; selection into the complete-case analysis was therefore characterized directly. Complete-case analysis itself may introduce selection bias when data are not missing completely at random; the comparisons in Table S1 characterize, but cannot eliminate, this limitation. Pregnancies with biochemistry were slightly older, sampled a few days later, more often conceived by IVF/ICSI, and less often had a high-risk NIPT call than those without, whereas BMI and FF were similar (Table S1); the cohort with biochemistry therefore represents a somewhat lower-risk and differently selected clinical population, and the biomarker estimates apply to women who undergo combined screening and NIPT in the same center. Similar observed FF distributions do not establish missing at random, and selection bias in the biomarker coefficients cannot be excluded. The interval between biochemistry and the cfDNA draw was measured from the date of the first-trimester examination at which the biochemistry was documented, not from a verified date of serum sampling, and in 22% of the primary cohort this date was after the NIPT draw; the biomarker estimates were unchanged when the analysis was restricted to pregnancies documented on or before the draw. FF was reported by multiple providers using different methods, and although platform was modeled and the β-hCG results were consistent within platform-restricted subsets, residual between-platform heterogeneity in the FF scale cannot be excluded and may contribute to the instability of the PAPP-A estimate. The mode of conception was recorded as documented IVF/ICSI or not, so that the reference group may include undocumented assisted conceptions. The numbers of low-FF and no-call events were too small for adjusted analysis, and the question of PAPP-A at the extreme of the distribution remains open. Placental growth factor (PlGF), a more direct marker of early placental mass and vascularization, was not measured in this cohort, so that the placental hypothesis could be examined only through PAPP-A and β-hCG. Fetal karyotype and pregnancy outcome were documented in a minority of pregnancies, precluding estimates of test performance and of placental outcomes, and excluding pregnancies with a high-risk NIPT call does not establish euploidy in the remaining pregnancies. Finally, the population was that of a referral center, and the tested women were self-selected, which limits the generalizability of the screening-strategy analysis.

## 5. Conclusions

In a clinically phenotyped NIPT cohort, cfDNA fetal fraction was independently associated with maternal BMI, documented IVF/ICSI, and testing platform; no independent linear association with gestational age was identified over the whole sampling range, although FF increased with gestational age within the 10–14-week window in the larger baseline cohort. Among first-trimester serum markers, free β-hCG was independently and consistently associated with fetal fraction, and the association remained similar after exclusion of pregnancies with a high-risk NIPT call, documented multiple gestation and documented IVF/ICSI, as well as in platform- and timing-restricted sensitivity analyses; PAPP-A across its normal range showed a weaker and less consistent association. These findings are compatible with, but do not establish, a closer relationship of fetal fraction to syncytiotrophoblast endocrine activity than to the processes reflected by circulating PAPP-A. Whether very low PAPP-A identifies pregnancies at higher risk of test failure could not be assessed with the few events available and remains a hypothesis for larger studies. As a secondary observation, the cohort documents the shift in NIPT in this setting from a contingent second-tier test to broad first-line use, with a corresponding fall in the high-risk call rate.

## Figures and Tables

**Figure 1:**
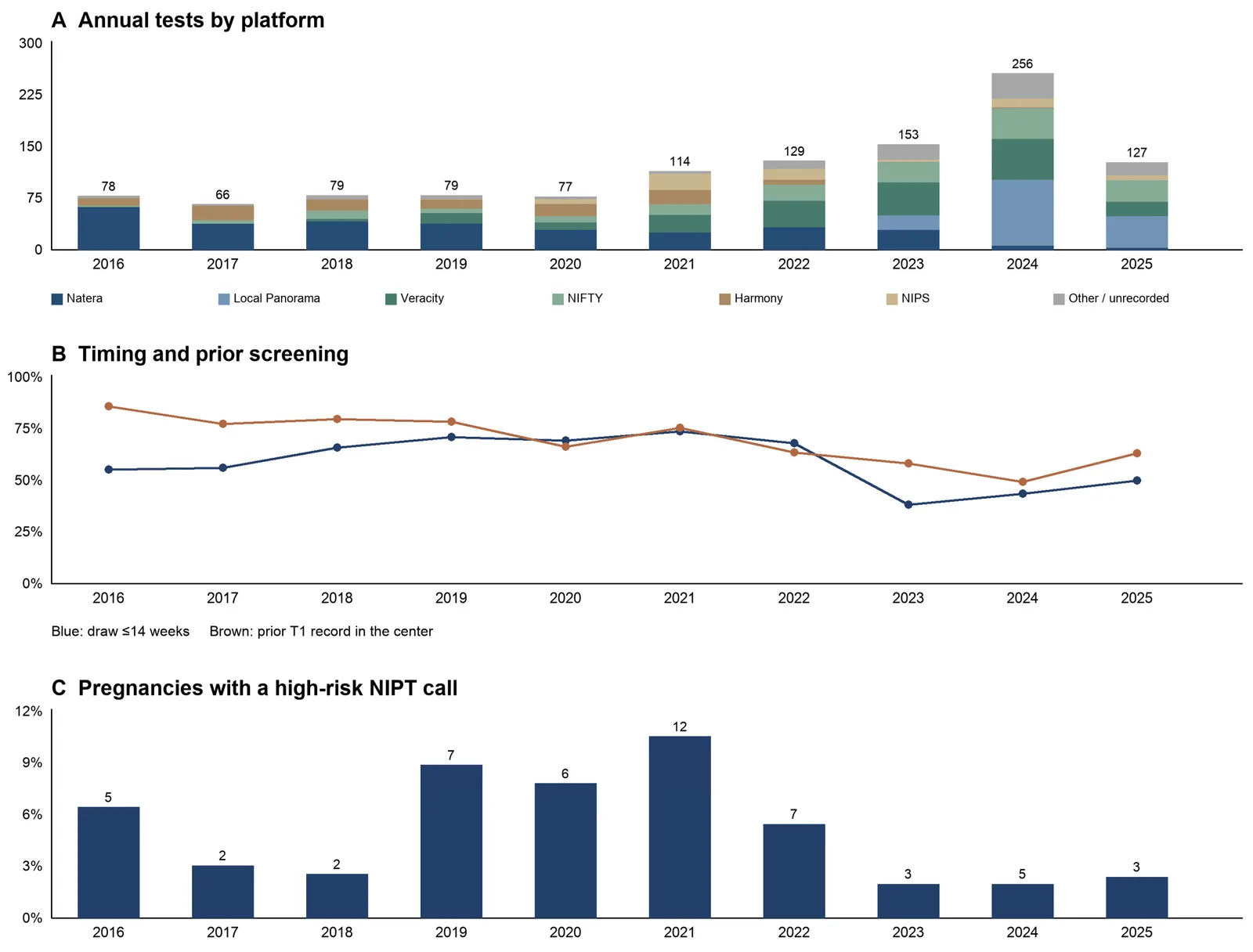
Descriptive NIPT trends by year of first blood draw, 2016–2025: (**A**) annual pregnancy counts by platform category; (**B**) proportion drawn at ≤14 weeks among pregnancies with valid gestational age (9–40 weeks), and proportion with a prior T1 record in the center among all pregnancies tested; (**C**) proportion with a high-risk NIPT call, with the number of high-risk calls above each bar. Four pregnancies with a first draw outside 2016–2025 (three in 2015 and one without a valid draw year) are omitted from this annual display.

**Figure 2:**
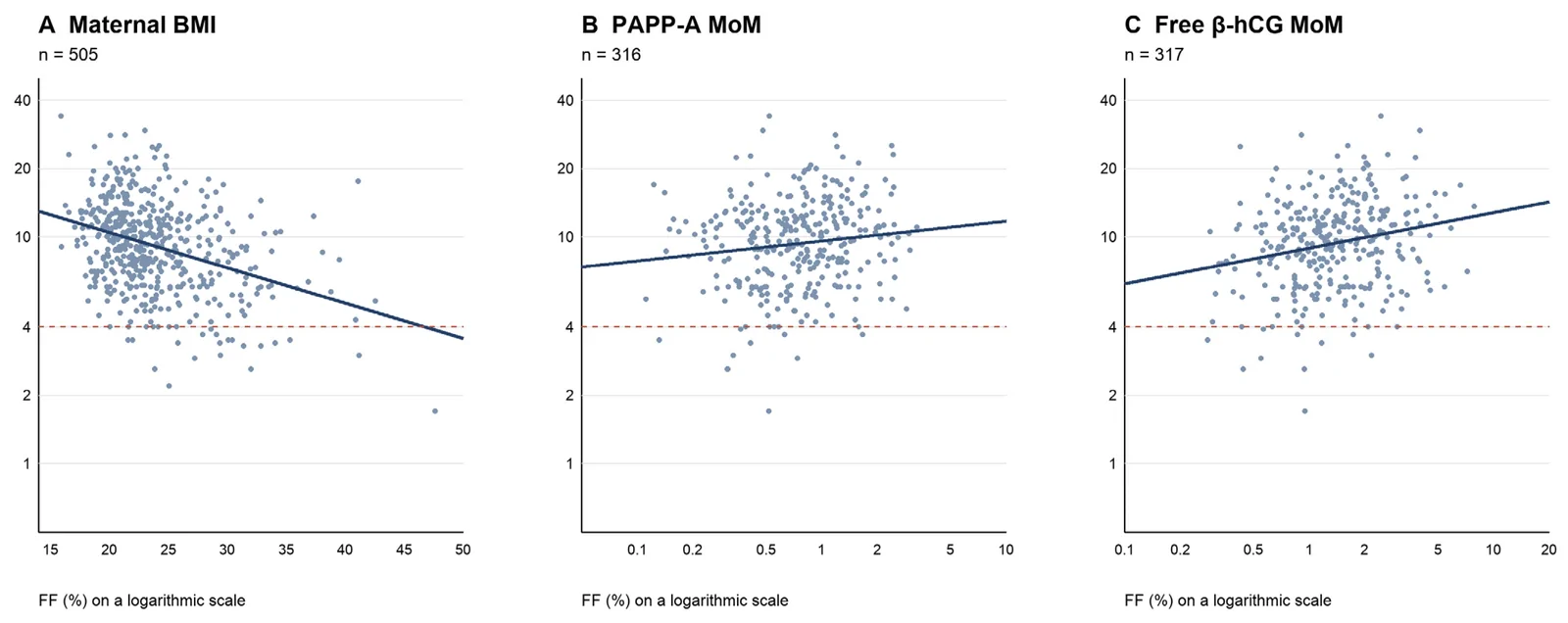
Fetal fraction (FF, logarithmic scale) in relation to (**A**) maternal BMI, (**B**) PAPP-A MoM and (**C**) free β-hCG MoM in the linked cohort. Biomarker axes are logarithmic. Solid lines are unadjusted linear fits of ln(FF) against BMI or ln(marker MoM); dashed lines mark FF = 4%. Populations are BMI *n* = 505, PAPP-A *n* = 316, and free β-hCG *n* = 317, as defined in Section 3.3.

**Figure 3:**
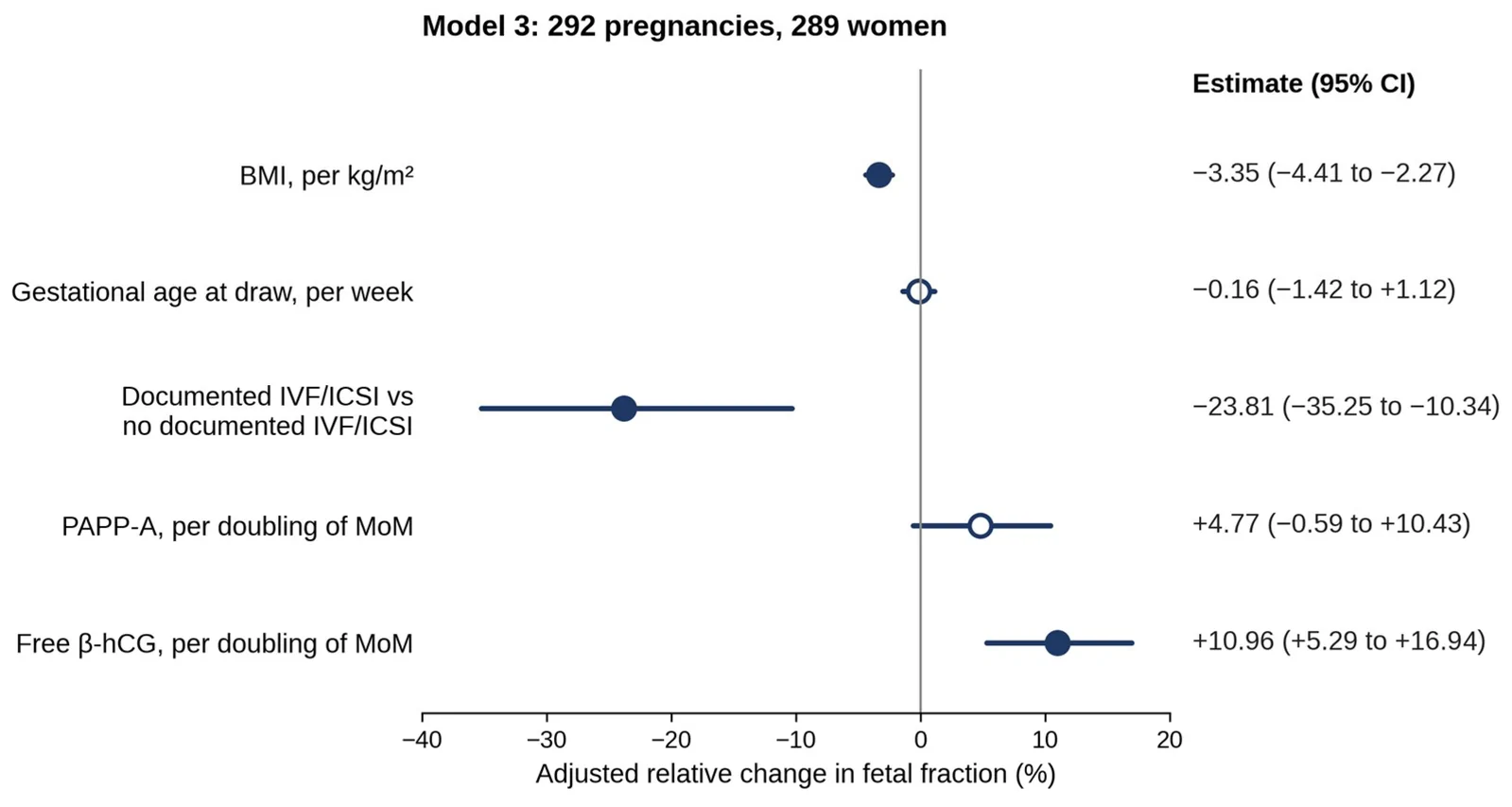
Adjusted relative percentage change in fetal fraction with woman-clustered 95% confidence intervals for selected Model 3 coefficients (*n* = 292): BMI per kg/m^2^, gestational age at draw per week, documented IVF/ICSI vs. no documented IVF/ICSI, and PAPP-A and free β-hCG per doubling of MoM. Filled markers indicate *p* < 0.05.

**Table 1:** Characteristics of the linked cohort (pregnancies with first-trimester screening in the center, first cfDNA draw per pregnancy).

Characteristic	*n* Available	Value
Maternal age at blood draw, years	759	33.9 (30.4–37.9)
Body mass index, kg/m^2^	539	22.9 (20.9–25.9)
BMI ≥ 30 kg/m^2^	539	56 (10.4%)
Smoking	758	98 (12.9%)
Documented IVF/ICSI	759	43 (5.7%)
Documented multiple gestation	760	8 (1.1%)
Gestational age at blood draw, weeks	752	13.3 (11.7–15.4)
Blood draw ≤ 14 weeks	752	460 (61.2%)
PAPP-A, MoM	338	0.75 (0.47–1.18)
PAPP-A < 0.4 MoM	338	59 (17.5%)
Free β-hCG, MoM	339	1.34 (0.89–2.09)
Nuchal translucency recorded	760	426 (56.1%)
Combined-test risk for trisomy 21 available	760	606 (79.7%)
Combined-test risk 1 in 100 or higher (≥1%)	606	38 (6.3%)
Male fetus (cfDNA)	704	386 (54.8%)
Fetal fraction, %	714	9.1 (6.7–12.3)
Fetal fraction < 4%	714	27 (3.8%)
High-risk NIPT call (T21/T18/T13)	760	38 (5.0%)
No-call result	760	10 (1.3%)

Values are median (interquartile range) or *n* (%). MoM, multiple of the median. Maternal weight was recorded in 541 pregnancies; valid BMI was available in 539.

**Table 2:** Cell-free DNA platforms, fetal fraction reporting and test failure (first draw per pregnancy, *n* = 1162).

Platform Category	*n*	Years of Blood Draw	FF Reported	FF, % Median (IQR)	FF < 4%	No-Call	High-Risk Call
Panorama (Natera, external)	304	2016–2025	95.4%	8.6 (6.4–11.0)	12/290 (4.1%)	8	18
Panorama (local)	163	2023–2025	98.2%	8.1 (5.6–10.5)	11/160 (6.9%)	2	2
Veracity	223	2018–2025	94.2%	9.4 (6.9–12.0)	6/210 (2.9%)	0	13
NIFTY	181	2015–2025	92.3%	10.6 (7.3–16.3)	11/167 (6.6%)	0	8
Harmony (Ariosa)	110	2015–2024	87.3%	9.4 (8.1–12.3)	1/96 (1.0%)	1	6
NIPS	71	2019–2025	87.3%	10.0 (7.0–14.4)	2/62 (3.2%)	0	2
Other or unrecorded	110	2016–2025	90.9%	9.2 (6.0–11.5)	2/100 (2.0%)	1	3

FF, fetal fraction; IQR, interquartile range.

**Table 3:** Fetal fraction by category of maternal BMI (baseline cohort, *n* = 467).

BMI, kg/m^2^	*n*	FF, % Median (IQR)	FF < 4%, *n* (%)
<18.5	26	11.5 (9.2–13.0)	0 (0.0%)
18.5–24.9	306	10.0 (7.6–12.8)	2 (0.7%)
25.0–29.9	90	8.6 (6.0–11.6)	6 (6.7%)
30.0–34.9	35	7.2 (5.5–8.6)	3 (8.6%)
≥35.0	10	6.1 (5.3–8.3)	1 (10.0%)

Baseline cohort: pregnancies with FF, a valid gestational age at draw, a valid BMI, and complete baseline covariates (Figure S1). Denominators of the univariable analyses in the text differ because the BMI–FF correlation required only FF and BMI, and the biomarker correlations required FF, a valid gestational age, and the marker concerned.

**Table 4:** Hierarchical linear regression models for log fetal fraction in the linked cohort (complete cases, *n* = 292). Coefficients from the fully adjusted Model 3 are expressed as percentage change in fetal fraction with 95% confidence intervals; standard errors are cluster-robust by woman. All models also include maternal age, smoking, fetal sex, and platform (seven categories).

Model 3 Coefficient	% Change in FF (95% CI)	*p*
BMI, per kg/m^2^	−3.35 (−4.41 to −2.27)	<0.001
Gestational age at draw, per week	−0.16 (−1.42 to +1.12)	0.808
Documented IVF/ICSI vs. no documented IVF/ICSI	−23.81 (−35.25 to −10.34)	0.001
PAPP-A, per doubling of MoM	+4.77 (−0.59 to +10.43)	0.082
Free β-hCG, per doubling of MoM	+10.96 (+5.29 to +16.94)	<0.001
Variance explained	R^2^ (95% bootstrap CI)	
Model 1 (maternal and technical covariates)	0.180 (0.131–0.307)	
Model 3 (+PAPP-A + free β-hCG)	0.238 (0.184–0.366)	
Joint contribution of both biomarkers	0.057 (0.017–0.121)	
Sequential ΔR^2^, PAPP-A then β-hCG	0.011, then 0.046	
Sequential ΔR^2^, β-hCG then PAPP-A	0.048, then 0.010	
Semi-partial R^2^, PAPP-A	0.010 (0.000–0.041)	
Semi-partial R^2^, free β-hCG	0.046 (0.011–0.098)	
Partial R^2^, PAPP-A	0.012 (0.000–0.056)	
Partial R^2^, free β-hCG	0.057 (0.014–0.126)	

Semi-partial R^2^, unique contribution of each biomarker given all other terms in Model 3; partial R^2^, semi-partial R^2^ divided by 1 − R^2^ of the model without that biomarker. Bootstrap CI, woman-level percentile cluster bootstrap with 5000 replicates.

**Table 5:** Sensitivity analyses for the biomarker coefficients of Model 3 (percentage change in fetal fraction per doubling of MoM, 95% CI). All models include BMI, gestational age at draw, maternal age, smoking, documented IVF/ICSI, fetal sex, and platform (the IVF/ICSI term is omitted in the analysis excluding IVF/ICSI).

Analysis	*n*	PAPP-A	*p*	Free β-hCG	*p*
Main model (Model 3)	292	+4.77 (−0.59 to +10.43)	0.082	+10.96 (+5.29 to +16.94)	<0.001
Excluding high-risk NIPT calls (T21/T18/T13)	286	+6.39 (+1.00 to +12.07)	0.020	+11.61 (+5.86 to +17.67)	<0.001
Excluding documented multiple gestation	287	+5.34 (−0.05 to +11.02)	0.052	+10.83 (+5.09 to +16.88)	<0.001
Excluding documented IVF/ICSI	267	+4.55 (−1.14 to +10.57)	0.119	+12.93 (+6.79 to +19.42)	<0.001
Excluding NIFTY	252	+1.42 (−3.95 to +7.08)	0.611	+10.36 (+4.28 to +16.81)	<0.001
SNP-based assays only	140	+3.41 (−3.24 to +10.51)	0.321	+8.55 (+0.80 to +16.90)	0.030
Excluding NIFTY and high-risk calls	246	+3.13 (−2.27 to +8.82)	0.260	+11.16 (+4.95 to +17.75)	<0.001
SNP-based assays, excluding high-risk calls	138	+3.04 (−3.92 to +10.50)	0.399	+8.53 (+0.76 to +16.91)	0.031
First-trimester examination documented on or before NIPT draw	228	+3.09 (−2.94 to +9.48)	0.321	+12.53 (+6.04 to +19.41)	<0.001
First-trimester examination documented 0–28 days before or on the day of the draw	179	+1.11 (−5.23 to +7.88)	0.737	+13.19 (+5.38 to +21.58)	<0.001

MoM, multiple of the median; SNP, single-nucleotide polymorphism. Documented multiple pregnancies in the main model, *n* = 5; documented IVF/ICSI conceptions, *n* = 25; high-risk NIPT calls, *n* = 6.

**Table 6:** NIPT results according to the prior first-trimester combined-test risk for trisomy 21 (first draw per pregnancy, *n* = 902).

Combined-Test Risk	*n*	High-Risk Call, T21	High-Risk Call, Any Trisomy	No-Call	FF, % Median
1 in 10 or higher	21	12 (57.1%)	13 (61.9%)	0 (0.0%)	9.4
1 in 11 to 1 in 50	13	3 (23.1%)	3 (23.1%)	0 (0.0%)	10.6
1 in 51 to 1 in 100	15	3 (20.0%)	5 (33.3%)	0 (0.0%)	8.5
1 in 101 to 1 in 300	67	4 (6.0%)	5 (7.5%)	3 (4.5%)	9.0
1 in 301 to 1 in 1000	131	2 (1.5%)	4 (3.1%)	1 (0.8%)	9.4
Below 1 in 1000	655	5 (0.8%)	8 (1.2%)	6 (0.9%)	9.0
Total	902	29 (3.2%)	38 (4.2%)	10 (1.1%)	9.1

High-risk call, high-risk NIPT result as reported by the laboratory (not confirmed aneuploidy).

## Data Availability

The pseudonymized dataset is held by the Maternal-Fetal Medicine Center, Timișoara, and is not publicly available because of patient-confidentiality restrictions; aggregate data supporting the findings are available from the corresponding author on reasonable request.

## Supplementary Materials

The following supporting information can be downloaded at: https://www.mdpi.com/article/10.3390/life16101677/s1; Figure S1: Cohort flow diagram: NIPT records, distinct blood draws, pregnancies, linkage to the first-trimester record, and the nested complete-case populations of each analysis, with the number excluded at each step; Table S1: Comparison of linked pregnancies with and without first-trimester serum biochemistry; Table S2: Fetal fraction and gestational age at blood draw: adjusted analyses.
