# Supplementary material for: Maternal Characteristics, First-Trimester Biomarkers and Cell-Free DNA Fetal Fraction: A Single-Center NIPT Cohort over Ten Years
Source: Life (Basel). 2026 Oct 7;16(10):1677. doi: 10.3390/life16101677 (PMC13705701; doi:10.3390/life16101677)
Supplement: Supplementary file 1 [file life-16-01677-s001.zip › life-4583578-supplementary.pdf]

Supplementary Material

Maternal Characteristics, First-Trimester Biomarkers and Cell-Free DNA Fetal Fraction: A Single-Center NIPT Cohort over Ten Years — Life, ID life-4583578

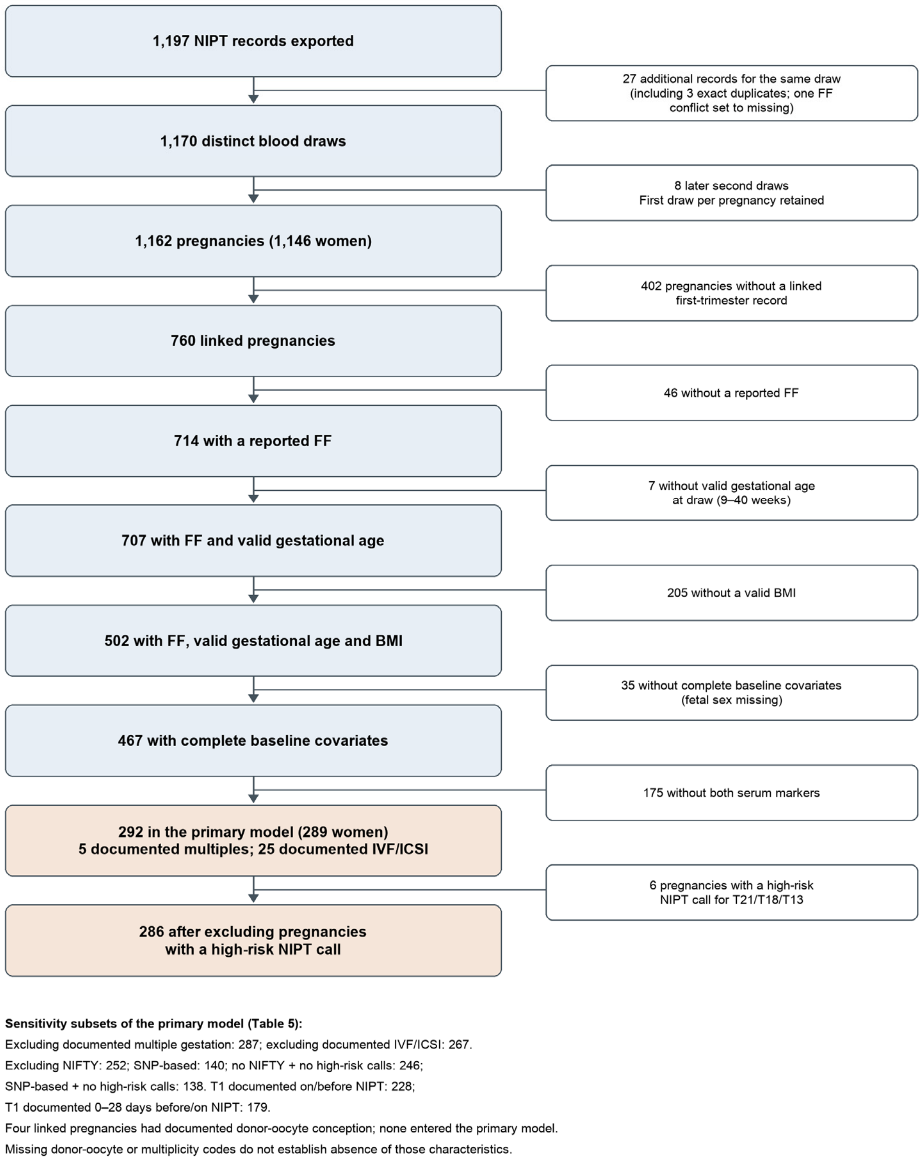

Figure S1. Cohort flow diagram: NIPT records, distinct blood draws, pregnancies, linkage to the first-trimester record, and the nested complete-case populations of each analysis, with the number excluded at each step.

Table S1. Comparison of linked pregnancies with and without first-trimester serum biochemistry.

Characteristic	With biochemistry (n = 338), value (n available)	Without biochemistry (n = 422), value (n available)
Maternal age at draw, years (median)	34.3 (n = 338)	33.7 (n = 422)
BMI, kg/m² (median)	22.9 (n = 336)	23.0 (n = 204)
Gestational age at draw, weeks (median)	13.7 (n = 337)	12.9 (n = 415)
Fetal fraction, % (median)	9.6 (n = 316)	8.9 (n = 398)
Documented IVF/ICSI	28/338 (8.3%)	15/422 (3.6%)
High-risk NIPT call (T21/T18/T13)	10/338 (3.0%)	28/422 (6.6%)

Medians (with the number of pregnancies in which the variable was available) and proportions (events/denominator). Biochemistry availability means both PAPP-A and free β-hCG are recorded. The records do not establish the missingness mechanism.

Table S2. Fetal fraction and gestational age at blood draw: adjusted analyses.

Analysis	n	% change in FF per week (95% CI)	p
Model 3, 9–40 weeks (primary)	292	−0.16 (−1.42 to +1.12)	0.81
Model 3, 10–14 weeks	155	−1.13 (−7.09 to +5.21)	0.72
Model 3, 11–13^{+6} weeks	128	−3.82 (−10.73 to +3.62)	0.30
Model 3, 10–20 weeks	263	−1.22 (−3.49 to +1.11)	0.30
Model 3, >14–20 weeks	108	−0.95 (−5.56 to +3.88)	0.69
Model 3, >20–30 weeks	26	+5.47 (−11.03 to +25.01)	0.53
Model 3 with separate slopes before/after 14 weeks	292	– (Wald test of the two slopes, p = 0.85)	0.85
Baseline model (Model 1 covariates), 10–14 weeks	283	+5.88 (+1.84 to +10.08)	0.004
Baseline model, 10–14 weeks, excluding high-risk calls	268	+5.26 (+1.04 to +9.66)	0.014
Baseline model, 10–14 weeks, restricted to the 155 pregnancies of the biochemistry model	155	+0.50 (−5.58 to +6.98)	0.88

Model 3: BMI, gestational age, maternal age, smoking, documented IVF/ICSI, fetal sex, platform, log PAPP-A MoM and log free β-hCG MoM. Baseline model: the same covariates without the biomarkers, in the baseline cohort. Unadjusted Spearman correlation across 9–40 weeks: $\varrho = 0.03$, n = 707, p = 0.35.
